# Current Advances in 3D Dynamic Cell Culture Systems

**DOI:** 10.3390/gels8120829

**Published:** 2022-12-16

**Authors:** Xin Huang, Zhengxiang Huang, Weidong Gao, Wendong Gao, Ruiying He, Yulin Li, Ross Crawford, Yinghong Zhou, Lan Xiao, Yin Xiao

**Affiliations:** 1 School of Mechanical, Medical and Process Engineering, Center of Biomedical Technology, Queensland University of Technology, Brisbane, QLD 4059, Australia; 2 School of Biomedical Sciences, The University of Queensland, St Lucia, QLD 4072, Australia; 3 College of Chemistry and Chemical Engineering, Hubei University, Wuhan 430062, China; 4 The Key Laboratory for Ultrafine Materials of Ministry of Education, State Key Laboratory of Bioreactor Engineering, Engineering Research Center for Biomedical Materials of Ministry of Education, School of Materials Science and Engineering, East China University of Science and Technology, Shanghai 200237, China; 5 Australia-China Centre for Tissue Engineering and Regenerative Medicine, Queensland University of Technology, Brisbane, QLD 4059, Australia; 6 School of Dentistry, The University of Queensland, Herston, QLD 4072, Australia; 7 School of Medicine and Dentistry, Griffith University, Gold Coast, QLD 4222, Australia

**Keywords:** 3D cell culture, mechanical stimulation on cell behavior, bioreactor, microcarrier, organ-on-a-chip

## Abstract

The traditional two-dimensional (2D) cell culture methods have a long history of mimicking in vivo cell growth. However, these methods cannot fully represent physiological conditions, which lack two major indexes of the in vivo environment; one is a three-dimensional 3D cell environment, and the other is mechanical stimulation; therefore, they are incapable of replicating the essential cellular communications between cell to cell, cell to the extracellular matrix, and cellular responses to dynamic mechanical stimulation in a physiological condition of body movement and blood flow. To solve these problems and challenges, 3D cell carriers have been gradually developed to provide a 3D matrix-like structure for cell attachment, proliferation, differentiation, and communication in static and dynamic culture conditions. 3D cell carriers in dynamic culture systems could primarily provide different mechanical stimulations which further mimic the real in vivo microenvironment. In this review, the current advances in 3D dynamic cell culture approaches have been introduced, with their advantages and disadvantages being discussed in comparison to traditional 2D cell culture in static conditions.

## 1. Introduction

Since Harrison Ross first carried out in vitro cell culture using a sterile coverslip in 1906 [1], the era for cell culture began. Nowadays, the cell culture technique is one of the most common techniques in many fields of biomedical sciences, from basic research to large-scale industrial production of biological products. It offers an efficient approach to achieving different purposes without using animals.

To culture most of the cell types outside of a living body, artificial devices are usually required to allow the cells to adhere and grow. Glass devices such as coverslips were most commonly used in the first few decades of cell culture history [2]. Later, plasma-treated polystyrene was invented by the Falcon Plastics Company and showed excellent properties for cell adhesion and growth [3]. More recently, plasma-treated polystyrene has dominated the research consumer market with different configurations designed for various research purposes, such as flasks, dishes, and plates. These cell culture devices allow adherent cells to grow in a monolayer on a two-dimensional (2D) planar surface under static conditions. With the established techniques in cell seeding, subculture, cryopreservation, and harvesting, it is convenient to perform in vitro experiments, typically using a single cell type, in a 2D and static environment at a relatively low cost. Therefore, such 2D and static monoculture have become the major approach for cellular biology investigation and drug testing on the laboratory scale.

Other 2D culture devices, such as the transwell system [4], have been developed to satisfy the demand for studying cell interactions via co-culture of two types of cells and individual analysis of multiple cell types. The transwell system includes an insert with a microporous membrane and a traditional 2D cell culture plate. While one type of cell can be seeded on the microporous membrane, the other type can grow on the companion well, forming a co-culture system without direct contact between the two types of cells. This approach is widely used to study cell–cell interaction through paracrine or endocrine [4,5,6]. Alternatively, two types of cells can be seeded on two sides of the membrane, forming a direct cell-to-cell contact co-culture, which is suitable for investigating barrier-structured tissues, such as the intestinal barrier [7], the blood–brain barrier [8], the blood–placenta barrier [9], the alveolar–capillary barrier [10], and the vascular model containing endothelial and vascular smooth muscle cells [11]. In both non-contacting and contacting settings, two types of cells share the cell culture medium and the secretome with each other. Although the transwell system has been used extensively, certain limitations exist; for example, it does not allow the individual analysis of more than two cell types, it limits controlling the cell ratios in the setting, and it is relatively expensive.

Despite the convenience and the extensive use, these traditional 2D and static culture devices have been questioned recently regarding their in vivo relevance because in a living body, cells are grown in a three-dimensional (3D) and dynamic, rather than a 2D and static, environment. Studies suggest that cells cultured in a 2D [12,13,14,15] or static [16,17] environment may lose some cell-specific properties observed in vivo, including morphology, polarity, differentiation, and metabolic profile. Cells grown on a standard cell culture plate have a more flattened shape because of the static culture method and a single growth direction [18]. Cell structure changes can influence nuclear morphology, which may alter the transcription and translation of genes [19]. It is also shown that a single cell can only interact with the same type of cells surrounding it when grown in a culture plate, which may be detrimental to the differentiation process [20]. This can explain why many new drugs show effectiveness in 2D and static cell culture systems in vitro but fail in further clinical trials [21]. A more realistic and vivid cell culture system is considered to benefit pharmaceutical development and toxicity tests [22]. Despite the lack of relevance to the in vivo condition, 2D and static cell cultures have other limitations for industrial applications. For example, in the large-scale production of cell protein or stem cells, the 2D culture has some limitations, such as surface-to-volume ratio and a lack of monitoring and control of critically metabolic parameters [23].

New cell culture systems that allow cells to grow in a 3D environment have been developed to overcome these shortcomings in recent years. In addition, based on the 3D culture model, a dynamic cell culture system has been designed to provide a more reliable cell growth environment in vitro. Firstly, a dynamic cell culture system can offer a continuous dynamic environment which is able to mimic cell growth conditions in vivo to promote cell proliferation and differentiation [24]. Besides, the dynamic culture method can help researchers to study the effects of physical stimulation during cell growth [25]. The third beneficial effect of dynamic culture is that different research purposes can be satisfied by adjusting the frequency, range, and period of the dynamic model [24]. The current 3D culture systems can be mainly categorized as (a) static 3D culture systems such as an extracellular matrix (ECM)-mimicking scaffold, which provides an ECM-like 3D environment for cell growth; (b) microcarrier and bioreactor-based systems, which provides a high surface-to-volume ratio suitable for large-scale production in a 3D and dynamic environment; and (c) organ-on-a-chip, a system combining various settings of cell culture platforms and microfluidics devices, which can provide 3D and dynamic environments and allow for multicellular co-culture and individual cell type analysis. With the development of such new cell culture devices, the application expands from culturing cells in vitro to serving as a platform to simulate the in vivo cellular interplay of specific organs/tissues. This review summarizes the effects of a 3D environment and dynamic mechanical force on cell behaviors and function. Meanwhile, the specification and application of the recently developed cell culture systems, with their advantages and disadvantages, are discussed, and the design of 3D and dynamic cell culture devices is proposed for future advances.

## 2. Effects of 3D and Dynamic Culture Environment on Cell Behaviors

In a traditional 2D cell culture system, the lack of a suitable 3D background environment and structural framing will influence cell behavior. For instance, normal epithelial cells always lose their differentiation ability and perform like cancer cells when they grow as 2D monolayer cells. Cells growing in a 3D environment show different behaviors in various aspects: morphology [26], proliferation [27], function [28], etc. In addition to the 3D environment, cells in vivo live in a dynamic environment that encounters continuous mechanical stress derived from the blood flow, interstitial flow, and body movement. Currently, regardless of the 2D or 3D environment, most cell culture approaches can only enable the cell to grow in a static environment without mechanical stimulation. Cells growing in static environments lack mechanical stimulation, which is an indispensable factor in modulating cell behaviors along with chemical stimulation. Cells in vivo can experience multiple mechanical force types, such as tension, pressure, and shear force, which significantly change cell behaviors. For example, myocardial cells grow under periodic tension from heartbeats [29]. Hemocytes, as a part of blood, flow across different blood vessels and are under shear force all their lifetime [30]. The transitional epithelium of the bladder receives pressure from the storage of urine [31]. Therefore, it is necessary to understand the different behaviors of cells growing in 2D and 3D environments and between cells under static and dynamic conditions.

### 2.1. Cell Behaviors in 3D Culture Environment

#### 2.1.1. Cell Proliferation and Differentiation in 3D Culture

The influence of 2D and 3D cultures on cell proliferation has been extensively studied. In nearly all the cell lines, tumour cells showed a higher proliferation rate in 2D monolayer cell culture than in 3D culture [20]. Research proved that the expression of polarization and differentiation associated of tumour marks integrins (β1 and β4) is higher in tumour cells from 3D culture, which suggesting the proliferation, adhesion, and viability of tumour cells are impaired [32,33,34,35,36]. Similarly, it was reported that endometrial cancer cells growing in a 2D environment had less specific function and organization than in a 3D culture. In another study, a 3D culture system using amphiphilic polydepsipeptides (HYDROX) was found to promote the differentiation of induced pluripotent stem cells (iPSCs) into the hepatic cell. Meanwhile, *CYP3A4*, an important metabolic enzyme-gene, which mostly expressed in human liver, was upregulated in primary human hepatocytes cultured with HYDROX, and these cells showed higher activity compared with those cultured in the 2D system [37]. Cells culture methods also determined the expression of genes associated with cytoskeletal protein, contractility, and matrix remodelling [24]. It was reported that cells growing in a 2D environment showed higher expression of ECM proteins than in a 3D environment [38].

#### 2.1.2. Cell Apoptosis in Cancer Drug Test in 3D Culture

Research showed that, when exposed to anticarcinogen drugs, apoptosis is more likely to occur when breast cancer cells grow in a 2D monolayer culture rather than when they form a 3D cell aggregate [39]. That was mainly because, in 2D culture, the absorption of drugs was not dependent on the gradient of cells, as dead cells would disperse into the medium, and living cells would always come into contact with the anticarcinogen [40]. Conversely, when cancer cells aggregated into a spheroid, the interior partitions could not fully contact drugs like surface cells, which suggested that inner cells would not be sensitive to the drugs and steadily divide into new tumour cells [41]. In addition, 3D cancer cell spheroids can produce cancer cell-derived ECM [42,43,44].

#### 2.1.3. Cell Motion and Migration in 3D Culture

The migration of cells appears differently between 2D and 3D cultures, since cells may have more complex interactions when they form a 3D aggregate [38]. In 3D cell aggregate, inner cells could hardly migrate towards the outside mainly because their migration is hindered by surrounding cells [45]. This phenomenon is critical in investigating cancer metastasis and other disorders. For instance, culturing in basement membrane extract, fibroblasts migrate much faster in the 3D environment (about 1.3 times than in a 2D environment). Additionally, more migration-associated signal cascades could be found in 3D culture than that in 2D culture. It was reported that β1-integrin could send several interaction signals to epidermal growth factor receptors in 3D cell culture, a phenomenon missing in the 2D environment [46].

### 2.2. The Effects of Mechanical Force on Cell Behavior/Function

#### 2.2.1. Stretching

Stretching is a common biological phenomenon defined as cells being put into a lengthening position caused by muscular movement or external forces. The stretching here refers to not only muscular but also skin and neuro-guided movement. Cells from these tissues will be stimulated during the stretching process. Cell stretching plays essential roles in both cell proliferation and apoptosis, which depends on the magnitude, frequency, and duration of mechanical extension. For example, in the ulna-loading study, low-strength mechanical stimulation (4000 μ-strain) could relieve osteocyte apoptosis-resulted bone resorption. In contrast, high mechanical stress (8000 μ-strain) caused osteocyte apoptosis and micro-damage of bone tissues [47]. In the clinics, continuous stretching force with different magnitudes and duration has been widely used in orthodontic treatment. This kind of stretching could activate signaling pathways such as p38 MAPK, JNK, and ERK in the human periodontal ligament (PDL), promoting the expression and activity of alkaline phosphatase (ALP), which is an early stage marker of osteogenic differentiation [48]. In regular breath, the stretching of the diaphragm muscular can induce the expansion of alveolar cells during the breath [49]. In burn cases, patients usually wear pressure suits in case of the formation of scar tissues. That is because the stretching of the skin can form a relatively anoxic environment in the burned part and inhibit fibroblasts from producing collagen [50]. In addition, the pull of the muscle causes the excitation of sensory nerve terminals in the muscle spindle, resulting in the motor neuron exciting the impulse to transmit sequentially through the spinal nerve anterior root and spinal nerve to the muscle, causing an opposite directional stretching of the power, termed “myotatic reflex” [51].

#### 2.2.2. Compression

Compression here is defined as cells being squeezed, normally by external forces, to achieve a smaller size or a flatter shape. Compressive force plays a crucial part in the bone remodeling process. Compressive force may initiate osteoclastogenesis during orthodontic tooth movement (OTM) [52]. Consequently, an excessive compressive force would lead to finger-like bone fracture [53]. Besides, under compressive force, TNF-α expression would be induced in periodontal ligament fibroblasts during OTM, directly related to the elevated RANKL expression and consequently resulting in induced osteoclastogenesis [54]. Moreover, in MC3T3-E1 cells (a pre-osteoblast cell line), the osteogenic differentiation could be impaired by compressive force through the ClC-3 chloride pathway and the expression of EphB4 and ephrinB2 [26,55].

#### 2.2.3. Contraction/Relaxation

The contraction and relaxation are the biological forces generated by the intestinal tract which can be helpful to the digestion and absorption of the nutrients. It was reported that the contraction and relaxation of drosophila midgut could promote the transportation of calcium ions and differentiation of intestinal stem cells. Similarly, the human intestine exhibits the same movement to affect the differentiation of stem cells [56].

#### 2.2.4. Shear Stress

Shear stress is the biological force generated by the blood flow on the endothelium, the inner layer of the blood vessel. In vivo, the heterogeneous phenotypes of arterial endothelium cause changes in blood flow patterns. Pulsatile or steady laminar flow could stimulate the endothelial cell (EC) to secrete functional factors and suppress coagulation, supporting EC survival [57]. On the other hand, ECs chronically experience arrhythmic changes in haemodynamic forces and exhibit different a behavior by enhancing cellular turnover (proliferation and apoptosis) and facilitating the adhesion of monocytes onto endothelium [58]. This change suggests a higher risk of function loss and atherosclerotic plaque formation [59]. In an in vitro study, the flowing culture medium was generated to mimic the hemodynamic shear stress in blood to stimulate ECs. The shear stress could be transformed into biological signals through integrin, which would be received by phosphoinositide 3-kinase (PI3-kinase) to activate the downstream signaling pathways in ECs [60].

## 3. Current 3D and Dynamic Cell Culture Approaches

As mentioned in previous sections, compared with the traditional 2D/static culture, the 3D and dynamic cell culture can bridge the gaps between in vitro cell culture and animal models by providing a repeatable and controlled environment to mimic the conditions in vivo. To date, various 3D cell culture methods have been developed, aiming at mimicking the cell interaction in tissues and organs in vivo. The application of these systems has made it possible to research deeper biochemical and biomechanical signals [61]. The development of 3D dynamic cell culture systems mainly can be categorized into microcarrier- and bioreactor-based systems, and microfluidic systems have shed new light into cell culture technology to potentially replace the use of laboratory animal models. In this section, we will summarize the recent advances in methods for static 3D culture and 3D dynamic culture systems.

### 3.1. Static 3D Cell Culture Approaches

A considerately established 3D culture system in bioengineering can be beneficial in promoting cell behaviors, such as cell cytoskeleton organization, cell differentiation, cell proliferation, and gene expression. The static 3D culture approaches can be divided into scaffold-free and scaffold-dependant methods, which will be discussed in the following parts.

#### 3.1.1. Scaffold-Free 3D Static Cell Culture

The scaffold-free 3D static cell culture systems are defined as methods to culture cell populations as spheroids in a 3D and static environment without using a scaffold for cell adhesion and growth surface. The spheroid culture methods rely on a different technique to gather the cells together, forming a spheroid-like cell aggregate, which mainly includes the low adhesion surface modification method and the hanging drop method [62,63]. Recently, 3D bio-printing, microfluidic channels techniques, and magnetic cell levitation were also applied in spheroid culture methods [64,65,66,67].

- Low adhesion surface modification method.

Low adhesion surface modification generally adopts a relatively simple strategy to prevent cell attachment to the culture surface. Consequently, cells would have to attach and automatically generate 3D spheroids. This can be achieved by using several culture surface modification approaches. Traditionally, 0.5% poly-2-hydroxyethyl methacrylate (poly-HEMA) was used to modify 96-well plates to inhibit cells attaching to the plate surface [68], which generated 3D cell spheres in multiple cancerous or non-cancerous cell lines, such as T47D, MCF7, MCF7-ADR, and MDA-MB-435 cells. Similarly, 1.5% agarose can coat the cell culture disk [69]. Further, the microchip technique has been developed to regulate the size the of cell aggregate and keep the cell spheres for at least 2 weeks [70]. Additionally, microwell plates made of particular non-adhesive accelerator materials allow many cell spheres to grow on them simultaneously [71]. In addition, micropatterning utilizes the pattern zone to seed the cell aggregates and control the growth of cells [72]. Meanwhile, researchers also tried culturing embryonic stem cells (ESCs) as embryoid bodies (EBs) in a collagen type I gel (GEL). EBs would form cluster-like tissues in GEL with occasional hollow and clear boundaries [73].

- Hanging drop method.

Another culture method is droplets, in which cells will aggregate and form spheroid-like tissues by using the hanging drop technique. Specifically, in this technique [74], droplets of the cell suspension are placed on the lid of a culture dish. Then, the lid is carefully inverted and placed on the top of the culture dish, which contains a culture medium to keep an environment conducive to cell growth. In the top end of droplets, suspended cells will come together and form a mini 3D aggregate. It allows long-term cell survival and maintains cell phenotype [61]. Cells grown in this way would differentiate more evenly [38].

However, these two methods mentioned above have obvious drawbacks. The medium and gas exchange is limited to a small droplet, which may lead to cell death and necrosis in the central part of the cell aggregate [75,76]. Indeed, most cells in the center of droplets are quiescent without enough oxygen and nutrient support [39]. Additionally, instead of forming a simple aggregate, cells in vivo attach to their ECM to create a well-organized tissue structure. This suggests that scaffold-free methods are not ideal for simulating the in vivo conditions in vitro.

#### 3.1.2. Scaffold-Dependent 3D Static Cell Culture

The scaffold-dependent 3D static cell culture has been developed to provide an ECM-like environment. Unlike the automatically formed 3D cell aggregation described above, the scaffold-dependent culture system offers a 3D scaffold with surfaces for cell attachment and growth. In addition, the porosity of the scaffold could provide efficient oxygen, nourishment, and metabolism waste exchange between the inside area and outside of the culture environment [77,78]; therefore it is considered to benefit cell growth inside the scaffold. Currently, there are many types of scaffolds for cell culture. Researchers use different biological materials such as polymer, bioceramics, and bimetals, such as fibrin, bioactive glasses, and titanium [79] to form solid scaffolds, which are mainly categorized into four types as listed below.

- Natural ECM-derived scaffolds.

Natural-derived ECM could cause suitable conditions for cell growth in human tissues. Therefore, native ECM proteins are considered a suitable biomaterial that could induce beneficial cellular behaviors. For example, the early commercial wound healing product, using synthetic mesh conjugated with porcine collagen provided a temporary barrier between the wound bed and the air to protect the underlying cellular environment [80]. Recently, unique acellular materials derived from the urinary bladder and placenta matrix were enriched with growth factors to improve wound healing [81,82,83]. In a living body, cells are embedded within ECM, a 3D network mainly consisting of proteoglycans and fibrous proteins (collagens, elastins, fibronectins, and laminins) [84]. The ECM provides structural support to the cells and interacts with the cells in a biochemical way through cell surface receptors [84]. Biopolymers such as collagens or fibronectins, which are extracted from animal ECM, contain similar biochemical elements to actual tissues and organs, therefore, are capable of accelerating tissue regeneration. The biopolymer-based scaffold allows cells to seed into its highly open porous structures where cells would not flatten and maintain their microstructure. Besides, cells can adhere and elongate along the scaffold, which allows cell alignment and directed culture [41]. The scaffold may need to carry ECM to mimic the natural ecological niche cells inhabit. Although the ECM-based scaffold has been used in tissue regeneration of bone, skin, and cartilage, it can hardly meet all the tissue repair demands (biochemical property, elasticity, and porosity) at the same time [85], therefore is not used as frequently as synthesized scaffolds described below.

- Hydrogels scaffolds.

Hydrogels are hydrophilic and polymeric networks that can absorb a large amount of water [86]. Hydrogels can be synthesized with either natural raw materials (collagen, alginate, chitosan, hyaluronic acid, cellulose, etc.) or synthetic raw materials (polyethylene glycol, polyvinyl alcohol, and polyhydroxyethyl 2-methyl acrylate) composed or compounded to form hydrogels. Natural materials have excellent cell adhesion, hydrophilicity, biocompatibility, and bioactivity, as they are mainly derived from animals themselves [70]; synthetic materials possess excellent mechanical properties to be combined with stretching devices [87] for dynamic cell culture, making hydrogels a good in vitro platform to be applied, as well as having high reproducibility and a low biological impact on cells [70].

To harvest the cells from hydrogels, an enzymatic treatment to degrade the hydrogel is usually required [88], during which care must be taken not to disrupt the cell integrity.

Hydrogels, with their ECM-mimicking 3D meshwork and excellent water content [86] exhibit characteristics closer to in vivo conditions in terms of cell migration [87], proliferation [89,90] and transcriptome profiling [90,91]. For example, human ovarian cancer cells cultured in hydrogel show higher adhesion protein expression and higher resistance to chemotherapy than those cultured on the 2D surface [92]. In another study, hydrogel 3D culture relieved the senescence-related changes and sustained energy metabolism stability of the adipose-derived mesenchymal stem cells, which underwent senescent in the 2D culture [93]. Madin-Darby canine kidney (MDCK) renal epithelial cells could form hollow spherical cysts in hydrogel rather than flat monolayer sheets in a traditional 2D culture [46]. Besides serving as cell-culture platforms, hydrogels can load and release bioactive substances in a controlled manner to regulate cell functions [87]. These characteristics make hydrogel a good in vitro platform for fundamental biomedical studies.

Hydrogel is also a powerful tool in stem cell-based therapies. First, hydrogel allows stem cells to maintain an undifferentiated state over a long-term culture [87]. Second, after delivering the hydrogel-containing stem cells to the target site, the hydrogel can retain the cells and provide a microenvironment to improve viability and function [89]. In addition, hydrogel can be made injectable and is suitable for repairing irregular sites, such as bone defects [90]. Nowadays, different hydrogels are commercially available for various purposes, including drug delivery, wound dressing, tissue engineering, etc. [91].

To summarize, the hydrogel can provide the cells with a 3D and potentially dynamic environment, leading to higher in vivo relevance. However, certain limitations exist. For example, although it is possible to co-culture multiple cell types in a hydrogel setting, it is usually impossible to harvest and analyze them individually. Despite the ability to stretch in three dimensions, most hydrogels with excellent mechanical properties contain cross-linking agents that may cause death and mutation of cells or DNA [71]. In addition, some cells can only be cultured for relatively short periods due to problems with the diffusion of nutrients through the hydrogel [40].

- Synthetic polymer scaffolds.

Synthetic polymers such as polycaprolactone (PCL), polylactic acid (PLA), and polyurethane are frequently used as raw materials to fabricate scaffold [94]. Compared with natural and hydrogel scaffolds, synthetic polymer scaffolds have much stronger mechanical properties [95]. Therefore, they can be applied in conditions requiring specific mechanical strength (e.g., scaffold for bone tissue engineering) [96]. Synthetic polymer scaffolds have been widely used in tissue engineering to promote injury healing and provide mechanical support until the cells and newly formed tissue integrate with native tissue [97]. They could also serve as 3D cell culture models to investigate cell behavior and the underlying mechanisms [94,96,97]. The disadvantages of synthetic polymers are their physio-chemical properties which are not suitable for cell attachment and proliferation (e.g., the hydrophobicity of PLA hinders cell adhesion) [97]. It often needs surface modification to improve its affinity with cells, such as plasma treatment and surface coating with collagen, fibronectin, and vitronectin [94,95].

- Metal and ceramic scaffolds

Besides polymers, metals, and ceramics such as titanium, magnesium, and tricalcium phosphate are also frequently used as raw materials for fabricating scaffolds [98,99,100]. Compared with polymers, they could provide higher mechanical strength for a certain particular use, such as bone substitutes [100]. Ceramics are conducive to bone regeneration [98,99]. Tricalcium phosphate especially can mimic the constitution and structure of bone, and the associated ceramics 3D scaffold has been found to benefit bone tissue engineering by facilitating bone mineralization [99].

### 3.2. Current 3D Dynamic Cell Culture Systems and Applications

As explained in Section 2.2, despite the 3D environment, cells in tissue/organs sense continuous mechanical stimulation in vivo due to the fluidic flow and body movement, especially the force types such as stretching, compression, and shear stress. Currently, there are culture systems generating stretching force on cells and culture systems inducing vibration force on cells, such as loudspeaker-based, bioreactor-based, ultrasonic-based, and vibration cell culture systems [24,101]. In contrast, most of them are 2D culture systems that rely on a membrane to induce mechanical stimulation on the cells growing on it (except for bioreactor-based culture systems) [24]. To date, the most frequently used 3D dynamic culture systems can be divided into bioreactor and microcarrier-based culture systems and microfluidic device-based organ-on-a-chip systems [102,103].

#### 3.2.1. Bioreactor and Microcarrier-Based Culture System

**Figure 1:**
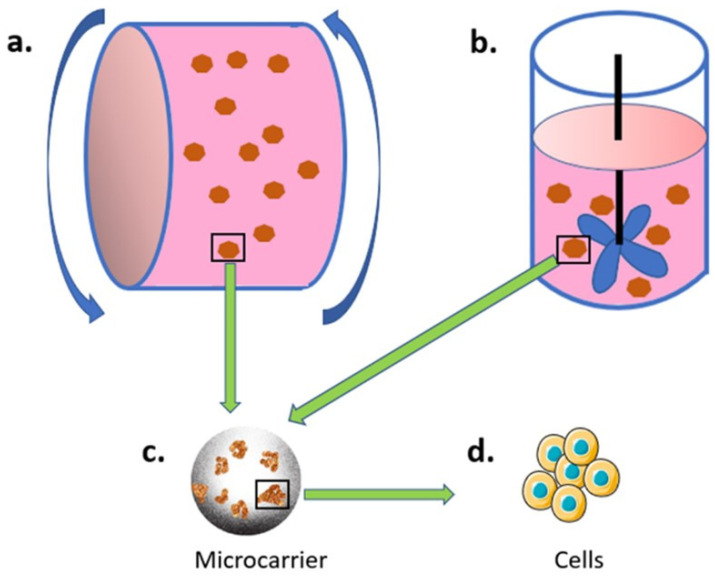
Bioreactor and microcarrier-based culture system. Microcarrier (**c**) are used for cell (**d**) adhesion, and the cell-loading microcarriers are then placed in either (**a**) rolling or (**b**) spinning bioreactors.

Researchers have recently used bioreactor-based systems such as spinner flask and rotating wall bioreactors to culture cells in a 3D dynamic environment [104,105]. These devices aim to create a dynamic environment for better nutrients exchange, homogenous oxygen gradient, and a small amount of shear force to the cells. While the spinner flask bioreactors achieve these by using a stirring bar in the middle and creating medium movement horizontally (Figure 1b), rotating wall bioreactors rotate and move the medium in a circulatory manner (Figure 1a). Using bioreactor systems, many indicators can be analyzed, such as the gas content of cells, which can be utilized for several purposes, including the measurement of oxygen content, bio-signal transduction, and vascular branching differentiation [106]. The influence of shear force in cell culturing also can be studied in this dynamic system [107]. Among these applications, the increase of cell proliferation and differentiation is generally considered the most important benefit of bioreactors.

Cell culture in the bioreactor depends on carriers for (adherent) cells to attach and grow. Currently, microcarriers are frequently used in bioreactor-based 3D dynamic cell cultures. Microcarriers are small beads with a diameter of approximately 100–300 μm, which are designed for cell attachment and growth in a 3D and dynamic environment (inside a bioreactor). The microcarrier was first developed by Van Wezel in 1967 [108], with a successful application on cell culture using anion exchange resin beads. The idea of the microcarrier cell culture is to have large surface areas in a relatively low volume that can be operated in a single unit, thus reducing the cost and simplifying the procedures of large-scale cell culture.

Currently, there are hundreds of microcarriers with different designs. These microcarriers can be categorized based on material and morphology. In terms of material, most microcarriers are made from either natural polymer (e.g., crosslinked dextran, collagen, gelatin, and cellulose) or synthetic polymers (e.g., polystyrene, polyacrylamide, and poly hydroxyethyl methacrylate), due to their good biocompatibility and reproducibility [109,110]. To facilitate cell attachment, growth and function, various surface modifications are also available, such as positive charging and ECM proteins coating [111]. In terms of morphology, the microcarriers can be classified into two categories, (1) solid with a smooth surface and (2) porous structure [109]. Solid microcarriers have good properties for cell adhesion and expansion, but the cells may be damaged by the shear stress and the collision between microcarriers. The porous structure further increases the surface area to volume ratio of the microcarrier but makes the oxygen and nutrient exchange more difficult. Cell culture using microcarriers typically occurs in a bioreactor, where a dynamic culture environment is created by agitation to keep the microcarriers suspended and distributed evenly. The product of the microcarrier cell culture could be either the cells or the secretome in the culture medium. An enzymatic assay, e.g., trypsin, is usually required to harvest the cells from microcarriers.

The most significant advantage of the microcarrier is that it economically suits the large-scale cell culture. A well-known example of microcarriers in large-scale production is the production of inactivated or live attenuated virus vaccines with the Vero cell line, which has been commonly used to produce many vaccines, including some SARS-CoV-2-based vaccines [112]. In addition, microcarriers are widely used in stem cell expansion for stem cell therapy [113,114,115], including the expansion of human mesenchymal stem/stromal cells (MSC) and induced pluripotent stem cells (iPSC). Stem cells expanded by microcarriers show advantages not only in the large-scale with a relatively low cost but also in the quality and reliability of the stem cells, including less heterogeneity [116] and better function in the downstream applications [117,118,119], compared to the ones expanded by the 2D cell culture systems. In addition, differences in morphology, surface markers, gene expression profiles, and secretome are observed between the microcarrier and conventional 2D systems in the stem cell culture [116,120]. The other characteristic of microcarrier cell culture is that cells can go through a bead-to-bead transfer [121], which simplifies the subculture process in the large-scale production of stem cells. In addition to large-scale production, microcarriers can also serve as a vehicle for cell delivery in tissue regeneration, especially in bone and cartilage tissue engineering. For example, injectable microcarriers have been developed to carry cells to repair and reconstruct irregular defects in a minimally invasive manner [122].

With material science advances, some microcarriers with novel functions have been developed recently. For example, microcarriers made by polygalacturonic acid polymer chains crosslinked with calcium ions can be dissolved in a solution containing EDTA and pectinase, which simplifies the cell harvest step without affecting the quality of iPSC culture [123]. A temperature-sensitive microcarrier coated with poly(N-isopropyl acrylamide) showing cell adhesion at 37 °C and detachment at 20 °C also allows easy cell harvest [124]. Similarly, a pH-responsive microcarrier composed of polyglycerol and poly(ethylene glycol) is stable at pH 7.4 and can be degraded at pH 6.0 while releasing highly viable cells [125]. In addition, microcarriers crosslinked by a redox-sensitive crosslinker showed faster cell detachment and higher cell recovery in the presence of reducing agents compared to the regular microcarriers [126,127]. All the above microcarriers with novel functions show some advantages in cell harvest, thus contributing to future applications in large-scale production and tissue engineering.

Microcarriers mainly contribute to the industrial field, including large-scale production of stem cells or secretome from the cells. These also serve as potential tools for stem cell delivery in tissue engineering. The morphology of microcarriers is the critical factor of large-scale cell expansion in the bioreactor, which could influence the fluent characteristic of cell carriers and further influence the cell’s behavior. However, traditional microcarrier manufacturing methods such as crosslinking, lithography, and emulsion drops make it impossible to manufacture carriers accurately with the desired structure. Some new manufacturing methods, such as 3D printing, should be considered to further optimize the design of microcarriers to create a better growth environment for cells. Although microcarriers can perform dynamic cell culture in a 3D environment, neither the dynamic parameters nor the cell growth can be well controlled, which weakens their application in basic biomedical research.

**Figure 2:**
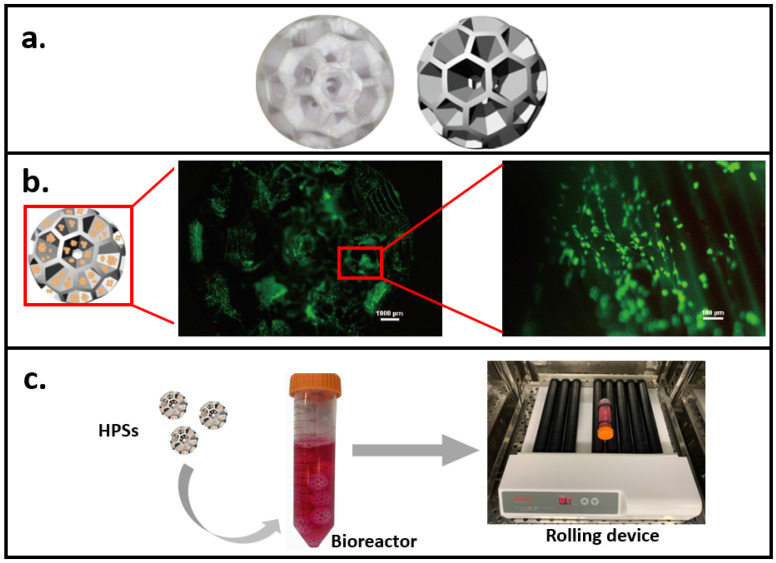
A novel 3D dynamic cell culture method. (**a**) 3D printed hollow porous scaffold (HPS) as cell carrier. (**b**) Fluorescent images showing cell (staining with FDA) growth on HPS. (**c**) The 3D dynamic culture system consists of the cell-seeded HPS, a bioreactor, and a rolling device.

In our recent study [128,129], based on a 3D printing technique, we printed hollow porous scaffolds (HPS) as cell carriers for 3D dynamic culture (Figure 2a). Considering biocompatibility and cytotoxicity, biodegradable aliphatic polymer PLA was chosen to be the printing material [130]. Two indexes for cell carrier design were considered, one is a porous structure with optimized surface shear stress; the other is inside velocity to guarantee gas and material exchange between the inside space of the carrier and the outside environment. Computational fluid dynamics (CFD) simulation modelling was used to analyze the surface shear stress (lower than 2 Pa (dynes/cm^2^)) of HPS [129], suggesting HPS should be able to protect cells from shear stress [131,132]. Inside velocity analysis [129] also showed HPS facilitated efficient nutrient/waste exchanges. Accordingly, cells could grow healthily and evenly on the surface of HPS, as shown in the FDA-staining results (Figure 2b). Subsequently, a novel 3D dynamic cell culture system was established (Figure 2c), by placing the cell-seeded HPSs in a bioreactor, which is then placed on a roller device to generate dynamic mechanical stimulation on cells.

This novel 3D dynamic culture system could serve as a potential research model in vitro, which proposing a new strategy to design a culture system via a combination of computer-aided design/modelling and experimental verification. The benefits of this culture system are that HPSs can provide sufficient growth area (one carrier equals to one well of 6-well plate) to culture a large amount of cells. Additionally, it is flexible and easy to separate cell carriers and it makes it possible to collect and analyze different type of cells at same time in cell co-culture. Moreover, based on the controllable rolling speed, this 3D dynamic system could satisfy different types of cells with their specific culture conditions. Additionally, in drug screening/testing, the sufficient medium exchange could facilitate drug delivery efficiency and drug effects.

#### 3.2.2. Microfluidic Cell Culture System (Organ-on-a-Chip) 

The microfluidic cell culture system is a group of devices to provide a dynamic culture environment (via generating fluidic shear stress) to the cells [129,133]. Like the commercial fluidic cell culture system, the microfluidic culture system consists of a pump, a carrier for cell growth, a connection system, and computer control systems. This system allows the cell culture medium to be continuously pumped (in a controlled manner) through the cell-growing carrier, therefore generating shear stress to regulate cell behavior and physiology. Therefore, Microfluidic cell culture systems are frequently used devices for biomedical investigations and drug screening. Unlike commercial fluidic cell culture systems, it can be customized for a particular application, especially the carrier can be customized with specific microarchitectures with the controlled shear flow to reconstitute the system in an in vivo environment.

**Figure 3:**
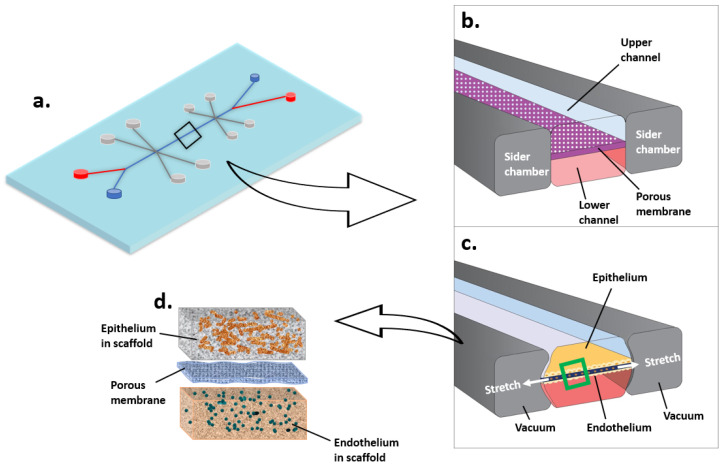
Microfluidic cell culture system (Organ-on-a-chip, taking lung as an example). (**a**) Lung on a chip. (**b**) The air flows through the upper channel. The blood flows through the lower channel. (**c**,**d**) Epithelium and endothelium grow in scaffolds on each side of the porous membrane. The stretch of membrane mimics lung breathing.

Based on the microfluidic device, the concept of organ-on-a-chip (Figure 3) has been developed in recent years. It describes a combination system with multiple cell types linked by microfluidics devices, aiming to mimic organ structure and function. The carrier within the organ-on-a-chip may include the previously mentioned transwell membrane and hydrogel, as well as the culture chamber in a specially designed shape and low-binding chambers for organoid culture. The organ-on-a-chip is usually fabricated with various materials, such as glass, silicon, and thermoplastics [134]. With the advanced technologies in material fabrication and microfluidics, currently, there are organ-on-chips under both physiological and pathological conditions for nearly every single organ, including lung [135], liver [136], heart and vessel [137], kidney [138], central nervous system [139,140], gut [141], pancreatic islet [142], adipose tissue [143], bone and musculoskeletal [144,145,146], periodontal tissue [147], female reproductive system [148], and tumor [149]. Furthermore, by combining multiple organ-on-chips in one setting, the “body-on-a-chip” or “multiorgan-on-a-chip” can be formed [134,150].

With the involvement of multiple cell types in a 3D environment and mechanical forces created by microfluidics, the organ-on-chips system may better mimic the in vivo condition compared to the traditional 2D and static models with a single cell type. For example, a dynamic liver-on-a-chip model consisting of hepatocytes, hepatic stellate cells, macrophages, and endothelial cells, showed better liver-like function compared to the monoculture of hepatocytes under static culture conditions, as indicated by higher albumin and urea synthesis and CYP3A4 protein expression [151]. Similarly, in an organ-on-a-chip system to study non-alcoholic fatty liver disease, co-culture of hepatocytes, hepatic stellate cells, and macrophages showed a more intense reaction to a high-fat medium than a hepatocytes monoculture, as indicated by enhanced inflammation, fibrosis, and reduced albumin production [152]. Therefore, organ-on-a-chip may be a valuable tool for mechanism studies and an efficient drug screening and toxicity testing platform. Indeed, Wang et al. engineered a heart-on-a-chip system using stem cell-derived cardiomyocytes from patients and confirmed the causal role of the gene Tafazzin mutation in the cardiomyopathy of Barth syndrome [153]. In another setting of organ-on-a-chip, Phan et al. cultured micro-tumor tissues with functional vascular structure and successfully identified anti-angiogenic and anti-tumor drugs from a small library of compounds [154]. Similarly, a high throughput liver toxicity screening system based on the liver-on-a-chip was designed recently [155]. Furthermore, with advances in the iPSC technology, a disease organ-on-a-chip model can be built using cells from the patient, enabling personalized drug screening in the future.

In addition, since the mechanical force is controllable by most microfluidic devices, organ-on-chips are powerful tools to investigate the effects of the dynamic environment (shear stress) on cell behaviors. For example, an intestine-on-a-chip device demonstrated significant differences in intestinal epithelial function, such as the expression of tight junctions and the production of mucus, in response to different levels of fluid shear stress [156]. In another study, a biochip showed that endothelial cells responded differently in cellular density, cell layer thickness, and gene expression profiles when stimulated by different levels of shear stress [157]. Both studies provide fundamental knowledge of the shear stress on cell biology and highlight the importance of the dynamic environment in the in vitro study.

Although the organ-on-a-chip system has advantages compared to conventional cell culture devices, there are limitations in certain aspects. First, it is a complex process to establish an organ-on-a-chip system, including multiple steps in fabrication, assembling, and cell seeding. The variations in each step may lower the repeatability. Second, the number of cells in the microfluidic device is usually small, which may not apply to high assays such as proteomics. Third, although an organ-on-a-chip mimics certain characteristics of the in vivo organs, such as cell type and the way cells are arranged, there is not yet strong evidence to prove whether it is functionally better than other systems, e.g., showing a better prediction of the in vivo drug efficacy. All these limitations must be considered when performing a study using the organ-on-a-chip system.

## 4. Conclusions and Future Remarks

**Figure 4:**
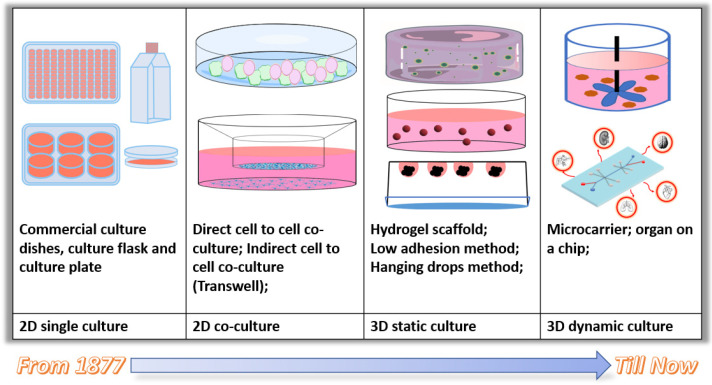
Evolution of the cell culture method.

The development of cell culture methods provides more choices to researchers for their specific study purpose (Figure 4, Table 1). 3D culture models that allow extra cell growth direction provides more opportunities for cell interaction, including signal delivery and secretor transportation and allows cells to get together from all directions, forming tissue-like structures. Meanwhile, dynamic culture models could mimic in vivo environments for cell growth with various stress stimulation.

However, current 3D culture models and dynamic culture models all have their limitations. Specifically, the aggregate culture system is beneficial for maintaining tissue function, but this system’s oxygen and nutrient support is insufficient [39]. The hydrogel technology system leads to nutrient diffusion [40] and the solid scaffold system lacks cell adhesion sites [41]. In addition, harvesting cells inside scaffolds is tricky, which limits their application in biomedical research. For the organ-on-a-chip system, the difficulty in setting up and the inability to produce large amounts of cells for classic molecular biology assays limit its application (Table 1). In the future, a new system that allows controllable 3D and dynamic environments, and co-culture of multiple cell types, is expected to be developed to mimic cellular interplay in tissues/organs in vivo, thereby satisfying the demands in biomedical research and industrial applications.

## Figures and Tables

**Table 1:** A summary of the functions of currently available cell carriers.

	Able to Provide a 3D Environment?	Able to Perform Static Culture?	Able to Perform Dynamic Culture?	Harvest a Large Number of Cells for Classic Molecular Biology Analysis?	Large-Scale Production?	Easy to Handle?	Cost?
Flask/plate/dish	N	Y	N	Y	Y	Easy	Low
Transwell system	N	Y	N	Y	N	Easy	High
Scaffold	Y	Y	Y	Y	N	Moderate	High
Low adhesion	Y	Y	N	N	N	Easy	Low
Hanging drops	Y	Y	N	N	N	Easy	Low
Microcarrier and bioreactor	Y	N	Y	Y	Y	Moderate	Moderate
Organ-on-a-chip	Y	Y	Y	N	N	Hard	Moderate

## Data Availability

Not applicable.
